# Associations of Three-Dimensional Anthropometric Body Surface Scanning Measurements and Coronary Artery Disease

**DOI:** 10.3390/medicina59030570

**Published:** 2023-03-15

**Authors:** Ning-I Yang, Li-Tang Kuo, Chin-Chan Lee, Ming-Kuo Ting, I-Wen Wu, Shuo-Wei Chen, Kuang-Hung Hsu

**Affiliations:** 1 Division of Cardiology, Department of Internal Medicine, Chang Gung University College of Medicine, Chang Gung Memorial Hospital, Keelung 204, Taiwan; 2 Division of Nephrology, Department of Internal Medicine, Chang Gung University College of Medicine, Chang Gung Memorial Hospital, Keelung 204, Taiwan; 3 Division of Endocrinology and Metabolism, Department of Internal Medicine, Chang Gung University College of Medicine, Chang Gung Memorial Hospital, Keelung 204, Taiwan; 4 Division of Gastroenterology and Hepatology, Department of Internal Medicine, Chang Gung University College of Medicine, Chang Gung Memorial Hospital, Keelung 204, Taiwan; 5 Laboratory for Epidemiology, Department of Health Care Management, Chang Gung University, Taoyuan 333, Taiwan; 6 Department of Health Care Management, Healthy Aging Research Center, Chang Gung University, Taoyuan 333, Taiwan; 7 Department of Emergency Medicine, Chang Gung Memorial Hospital, Taoyuan 333, Taiwan; 8 Research Center for Food and Cosmetic Safety, College of Human Ecology, Chang Gung University of Science and Technology, Taoyuan 333, Taiwan; 9 Department of Safety, Health and Environmental Engineering, Ming Chi University of Technology, New Taipei City 243, Taiwan

**Keywords:** three-dimensional anthropometrics, waist circumference, chest width, adiponectin, leptin, coronary artery disease

## Abstract

*Background and Objectives*: The relationship between three-dimensional (3D) scanning-derived body surface measurements and biomarkers in patients with coronary artery disease (CAD) were assessed. *Methods and Methods*: The recruitment of 98 patients with CAD confirmed by cardiac catheterization and 98 non-CAD patients were performed between March 2016 and December 2017. A health questionnaire on basic information, life style variables, and past medical and family history was completed. 3D body surface measurements and biomarkers were obtained. Differences between the two groups were assessed and multivariable analysis performed. *Results*: It was found that chest width (odds ratio [OR] 0.761, 95% confidence interval [CI] = 0.586–0.987, *p* = 0.0399), right arm length (OR 0.743, 95% CI = 0.632–0.875, *p* = 0.0004), waist circumference (OR 1.119, 95% CI = 1.035–1.21, *p* = 0.0048), leptin (OR 1.443, 95% CI = 1.184–1.76, *p* = 0.0003), adiponectin (OR 0.978, 95% CI = 0.963–0.994, *p* = 0.006), and interleukin 6 (OR 1.181, 95% CI = 1.021–1.366, *p* = 0.0254) were significantly associated with CAD. The combination of biomarker scores and body measurement scores had the greatest area under the curve and best association with CAD (area under the curve of 0.8049 and 95% CI = 0.7440–0.8657). Conclusions: Our study suggests that 3D derived body surface measurements in combination with leptin, adiponectin, and interleukin 6 levels may direct us to those at risk of CAD, allowing a non-invasive approach to identifying high-risk patients.

## 1. Introduction

Cardiovascular disease, in particular coronary artery disease (CAD), is one of the main causes of morbidity and mortality in the world [1]. Obesity is described as an independent risk factor for CAD [2] and is generally defined by an excess of body fat with the most commonly used anthropometric index being the body mass index (BMI) [3]. Obese individuals with the same amount of total body fat can have markedly distinct risk factor profiles [4], with abdominal fat having strong associations with CAD, mortality [5,6,7,8], and type 2 diabetes [9].

Visceral adipose tissue is an important endocrine organ, responsible for secreting hormones involved in a range of processes, e.g., control of sensitivity to insulin and inflammatory process mediators, and vascular hemostasis [10,11]. Biomarkers which play a role in insulin resistance and inflammation have been found to be associated with cardiovascular diseases. Leptin is an important link between obesity and the development of cardiovascular disease partially due to its effects on arterial pressure, formation of arterial thrombosis, aggregation of platelets, and on inflammatory vascular response [12]. Low adiponectin levels have also been found to be an independent risk factor for CAD [13]. The inflammatory biomarker C-reactive protein is positively correlated to the risk of cardiovascular events [14]. Interleukin 6 (IL6) and interleukin 8 have also been shown to play an important role in atherogenesis and atherosclerotic plaque destabilization [15,16]. It has also been demonstrated that the induction of the cytokine transforming growth factor beta-1 is associated with myocardial infarction [17].

A noninvasive three-dimensional (3D) scanning technology has been developed to obtain anthropometric measurements with many advantages over traditional methods, such as computed tomography scanners, X-rays, and bioelectrical impedance [18]. The aim of our study is to explore the association between CAD, biomarkers, and body measures with the use of 3D body scanning, providing more information to be used in clinical practice, epidemiological studies, and preventative medicine.

## 2. Materials and Methods

### 2.1. Study Subjects

From March 2016 to December 2017, a total of 98 patients found to have CAD as confirmed by cardiac catheterization exam at Chang Gung Memorial Hospital, Keelung, were recruited into our study CAD group. The same number of 98 sex- and age-matched patients presenting to our Department of Health Promotion and Examination were enrolled into the control group. Informed consent was obtained from all participants. This study protocol conforms to the ethical guidelines of the 1975 Declaration of Helsinki and was approved by the Institutional Review Board of Chang Gung Medical Foundation (201405148B0).

### 2.2. Anthropometrical Parameters

Three-dimensional body surface measurements were collected using a whole-body 3D laser scanner according to previously published methods. In addition to body weight, body height, and BMI, 35 measurements from four anatomical regions were made. The trunk region included the chest profile area, chest circumference, chest width (CW), waist profile area, waist circumference (WC), waist width, trunk volume, and trunk surface area. The head and neck region included the head surface area, head volume, head circumference, and neck circumference. The hip to the lower limb region included the hip profile area, hip circumference, hip width, left and right leg volume, left and right leg surface area, left and right calf circumference, left and right thigh circumference, and left and right leg length. The upper limb region included the left and right arm volume, left and right arm surface area, left and right arm length (RAL), left and right upper arm circumference, and left and right forearm circumference. The 3D laser scanning machine (LT3DCam) was built by Logistic Technology Company (LTC, Hsinchu, Taiwan), and was proven to have a high standard of accuracy due to the objective and comprehensive ways of measuring the human body surface. The standard procedure of measuring required the subject to remove all outer clothes except for underwear in preparation for scanning (women with bras in addition to pants) and to stand still on the stage for scanning (a total scanning time is about 10 s) [19]. The software system collected, realigned, constructed, and measured a subject’s whole-body digital stature and selected information. The measurement error of the 3D scanner in measuring the human body surface was checked; the error in the *x*- and *y*-axis was approximately 1 mm (1.2%), and in the *z*-axis it was less than 0.1 mm (0.2%) [20].

### 2.3. Data Collection

Upon recruitment, a questionnaire was given to acquire information on the following: date of birth; sex; occupation; education; marital status; history of cigarette smoking, alcohol drinking, and betel nut chewing; personal medical history (including hypertension, diabetes, heart disease, chronic kidney disease, liver cirrhosis, and chronic hepatitis). A medical chart review confirmed the answers provided. For those with no history of diabetes, a fasting blood glucose level was obtained. Diabetes was defined according to American Diabetes Association guidelines. For those without a history of hypertension, blood pressure was measured with a mercury sphygmomanometer on the left arm after the patient had been resting for 20 min in a seated position. Hypertension was defined according to the 2017 Hypertension Clinical Practice Guidelines (systolic blood pressure ≥ 140 mmHg, diastolic blood pressure ≥ 90 mmHg, or the use of antihypertensive medication) [21].

### 2.4. Laboratory Analysis

Venous blood was sampled overnight. Assays for high-sensitivity C-reactive protein were carried out in the Department of Laboratory Medicine, Keelung Chang Gung Memorial Hospital. Biomarkers including IL6, IL8, leptin, adiponectin, and transforming growth factor beta-1 were measured using commercially available enzyme-linked immunosorbent assays (Boster Biological Technology, Pleasanton, CA, USA).

### 2.5. Statistics

Two independent sample *t*-tests were used to compare differences between the continuous variables of the groups, and results were presented as the mean ± standard deviation (SD). The χ2 test was used to differentiate between the distribution of categorical variables, and results were expressed using frequencies and percentages between the groups. The 3D body surface measurements were screened using a two-sample *t*-test by comparing differences between CAD patients and controls. To avoid collinearity in the regression analysis, one body measurement with the lowest *p* value was selected from each anatomic dimension for subsequent multivariable analysis. A logistic regression model was used to determine the strength of the association between the selected body measurements and the presence of CAD. In addition to the forced-in sociodemographic variables, a backward model selection with *p* < 0.1 was used to determine variables, including lifestyle variables, to be retained in the regression model. The modulating effect was examined by comparing models with and without biomarkers while calculating the strength of association (odds ratio [OR]) between the body measurement combinations and CAD. In order to find associations with CAD in individual patients, biochemical and body shape variables that significantly differed between the non-CAD and CAD groups were further analyzed. This was done by calculating optimal cutoff values for continuous variables using a receiver operating characteristic (ROC) analysis. The statistical software used for the analyses in this study was SPSS 25.0 (IBM Corporation, Armonk, NY, USA).

## 3. Results

A total of 98 patients were recruited into each of the CAD group and control group over a period between March 2016 and December 2017. Baseline characteristics for the study participants are shown in Table 1. Both groups were matched for age and sex, with 76.53% of patients being male and 72.45% equal to or greater than 50 years of age in both CAD and control groups. More patients in the CAD group had a lower educational level (73.47% vs. 37.76%, *p* = 0.001). Among lifestyle variables, the CAD group had more smokers (56.12% vs. 36.73%, *p* = 0.0099)), more patients that did not consume coffee (54.08% vs. 35.71%, *p* = 0.0097), and more that did not exercise (52.04% vs. 37.76%, *p* = 0.044). As regards to risk factors, more patients in the CAD group had hypertension (50% vs. 1.02%, *p* < 0.0001) and diabetes (36.73% vs. 3.06%, *p* < 0.0001), and fewer patients in the CAD group had hyperlipidemia (20.41% vs. 39.8%, *p* = 0.0031).

Various biomarkers showed some differences between the CAD and control groups. (Table 2) Levels of HsCRP, IL6, IL8, and leptin were higher and adiponectin lower in the CAD group.

The 3D body surface scanning measurement results are shown in Table 3. The majority of measurements showed significant difference between the two groups, mainly with the CAD group having larger body measurements than controls. Associations between body measurements and biomarkers are presented in Table 4.

### Multiple Logistic Regression and ROC Analysis

The associations between different body measurements and biomarkers on the occurrence of CAD were further assessed using a multiple logistic regression model adjusted for sex, age, education, exercise, smoking, alcohol drinking and coffee consumption, hypertension, diabetes, and hyperlipidemia. It was found that CW (OR 0.761, 95% CI = 0.586–0.987, *p* = 0.0399), RAL (OR 0.743, 95% CI = 0.632–0.875, *p* = 0.0004), WC (OR 1.119, 95% CI = 1.035–1.21, *p* = 0.0048), leptin (OR 1.443, 95% CI = 1.184–1.76, *p* = 0.0003), adiponectin (OR 0.978, 95% CI = 0.963–0.994, *p* = 0.006), and IL6 (OR 1.181, 95% CI = 1.021–1.366, *p* = 0.0254 were significantly associated with CAD (Table 5).

**Figure 1:**
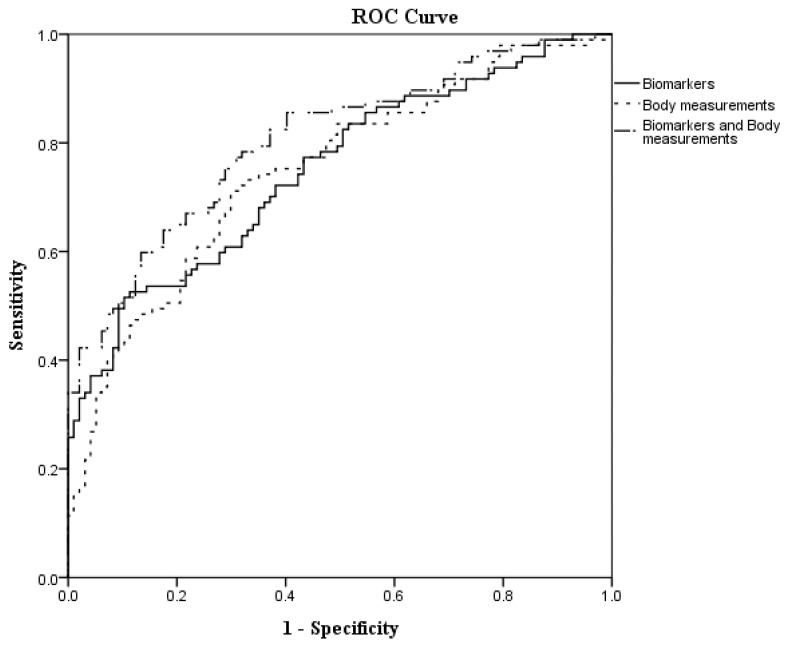
The receiver operating characteristic (ROC) curves to predict CAD.

The biomarker score, body measurement score, biomarker and body measurement score ware calculated based on the estimated values generated by the Table 5 model, and the scores were adjusted by risk factors. Receiver operating characteristic (ROC) curve analyses were adopted to estimate the predictive values of biomarker score, body measurement score and biomarkers combined with body measurements score for the occurrence of CAD. It was found that the combination of biomarker scores and body measurement scores had the greatest area under the curve and best association with CAD as shown in Figure 1 and Table 6. (Area under the curve of 0.8056, 95% CI = 0.7450–0.8662, *p* < 0.0001).

## 4. Discussion

Our study results show that lower educational level, no coffee consumption, physical inactivity, low adiponectin, high leptin, and high IL6 levels were associated with CAD. In terms of 3D body measurements, compared to the traditional BMI assessment, smaller CW and RAL with higher WC were also associated with CAD. In addition, the combination of biomarker scores and body measurement scores had the highest predictive value for CAD as shown with ROC analysis. These findings have given us a novel method for assessing the risk of those who may have CAD.

Inflammation contributes to CAD, among which IL6 plays an important role in atherogenesis and atherosclerotic plaque destabilization. IL6 is associated with vascular endothelial injury and tissue fibrosis, promotes angiogenesis, and increases vascular permeability [22]. Once IL6 levels are abnormally elevated, a series of pathological changes occurs including inflammatory injury, plaque formation and rupture, and thrombosis. Chronic exposure to IL6 also disturbs insulin action and body fat. Yet, despite having proinflammatory properties, IL6 also plays an important role in anti-inflammation. Enhanced fat oxidation occurs when IL6 is increased acutely, leading to improved insulin-stimulated glucose uptake with anti-inflammatory effects. With chronic secretion under obese conditions, these effects are not seen, probably due to the development of IL6 resistance [23]. In our study, it was found that IL6 was associated with CAD.

Adipose tissue is associated with CAD, abdominal adiposity causes development of adipose cells that are enlarged and dysfunctional [24]. These dysfunctional adipose tissues secrete pro-inflammatory biomarkers including prostaglandins, C-reactive protein, and cytokines such as interleukins and leptin with a decrease in adiponectin levels [25,26]. Leptin can cause vascular smooth muscle hypertrophy and oxidative stress, and stimulates vascular inflammation which may then lead to the development of type 2 diabetes mellitus, hypertension, atherosclerosis, and CAD [27]. Some studies have shown that increased leptin levels in plasma are associated with adverse outcomes in heart failure and CAD [28]. In CAD patients, higher serum leptin levels were significantly related to an increasing number of stenotic coronary arteries and arterial stiffness [29]. Another adipokine, adiponectin, also has important effects on the cardiovascular system. Its levels are negatively correlated with metabolic and cardiovascular disorders [30], with low levels having been shown to be an independent risk factor for cardiovascular disease [31,32]. In contrast to leptin, adiponectin levels are directly correlated with insulin sensitivity and inversely correlated with adiposity [33,34,35]. Certainly, as shown in our study population, the above mentioned adipokines were found to be associated with CAD.

By using a more accurate 3D body scanning method, we found that higher WC, lower CW and lower RAL were also associated with CAD. WC, which reflects abdominal obesity, has been suggested to be superior to BMI for CAD risk prediction [36], and this was similarly seen in our study. In addition to the important role it has in CAD, leptin has also been found to affect bone metabolism via both direct and indirect mechanisms [37]. Studies have shown that leptin resistance or insulin resistance as found in obesity may lead to poorer bone health [38,39]. Increased adiposity can also lead to decreased bone mass, affecting cortical bone more than trabecular bone [40,41]. These mechanisms may help explain the findings of shorter RAL associated with CAD in our study. Interestingly, CW was associated with CAD in our population. The thoracic cavity, when intact and closed, constrains the heart and lungs to a limited space, such that intrathoracic pressure changes throughout respiratory phases can have varying effects on cardiac function. Thus, one with a smaller chest width may have impaired pulmonary function or motion capacity of organs in the chest in addition to limitation of circulation flow rates. It has been shown that small whole heart volume predicts cardiovascular events in patients with stable chest pain [42]. During normal breathing, chest wall motion is determined by the displacement from respiration and the displacement by heart activity. There has been an interest in how chest wall motion provides information on the cardiorespiratory system with the design of different chest wall models [43]. A smaller CW may therefore also be an indicator that there is restricted cardiopulmonary displacement from cardiovascular impairment. Dynamic lung and chest wall compliance can be measured by the pressure–volume curve [44]. In fact, it has been documented that abdominal obesity preferentially depresses chest wall compliance resulting in a marked decrease in functional residual capacity and expiratory reserve volume [45]. This may very well explain the link between the high WC and lower CW we see associated with CAD in our population.

Faced with CAD being such an important cause of death worldwide, we sought to explore its associations with the more accurate method of 3D-derived body measurements and biomarkers in an attempt to gain more mechanistic insight.

### Limitations

As our study design was of a cross-sectional study, we are unable to infer causality from the results. The 3D body measurements were performed only at one point in time with no repeated estimations or data on changes over time. Our study population was of Chinese adults in a hospital setting so the results might not be applied to other ethnicities, age groups, or populations in the community. Therefore, it may be necessary to clarify these conclusions in further longitudinal studies and in a wider population.

## 5. Conclusions

In our study, 3D anthropometrics provide incremental information regarding associations of body surface measurements with CAD. It has been shown that shorter RAL and CW, and longer WC measurements combined with lower adiponectin and higher leptin and IL6 levels were associated with CAD. Although the precise mechanisms are far from clear, by combining non-invasive 3D body surface measurements together with biomarkers, we may in future be able to explore a different mechanistic approach to CAD, and non-invasively identify those with this condition in clinical practice, in addition to providing more information in epidemiological studies and preventative medicine.

## Figures and Tables

**Table 1:** Characteristics of study participants.

	No CAD		CAD		*p* Value
	n = 98		n = 98		
	Number	%	Number	%	
Sex					1
Male	75	76.53	75	76.53	
Female	23	23.47	23	23.47	
Age (years)					0.9971
<50	27	27.55	27	27.55	
50–54	18	18.37	17	17.35	
55–59	18	18.37	17	17.35	
60–64	19	19.39	21	21.43	
65≥	16	16.33	16	16.33	
Occupation					0.0954
Government employees	21	21.43	12	12.24	
Others †	57	58.16	71	72.45	
Housekeepers/students	20	20.41	15	15.31	
Educational level					0.001
Senior high school	37	37.76	72	73.47	
College and above	61	62.24	26	26.53	
Marital status					0.3453
Others	32	32.65	25	25.51	
Married	66	67.35	73	74.49	
Lifestyle factors					
Tobacco smoking					0.0099
No	62	63.27	43	43.88	
Yes	36	36.73	55	56.12	
Alcohol consumption					1
No	63	64.29	64	65.31	
Yes	35	35.71	34	34.69	
Tea consumption					0.668
No	49	50	45	45.92	
Yes	49	50	53	54.08	
Coffee consumption					0.0097
No	35	35.71	53	54.08	
Yes	63	64.29	45	45.92	
Betel nut chewing					0.1405
No	89	90.82	81	82.65	
Yes	9	9.18	17	17.35	
Exercise					0.0444
No	37	37.76	51	52.04	
Yes	61	62.24	47	47.96	
Diseases					
Hypertension					<0.0001
No	97	98.98	49	50	
Yes	1	1.02	49	50	
Diabetes					<0.0001
No	95	96.94	62	63.27	
Yes	3	3.06	36	36.73	
Hyperlipidemia					0.0031
No	59	60.2	78	79.59	
Yes	39	39.8	20	20.41	

CAD: coronary artery disease. †: Workers, businessmen, freelance workers, or service workers.

**Table 2:** Distribution of biomarkers between study groups.

	No CAD			CAD			*p* Value
	Means ± std.			Means ± std.			
HsCRP (mg/L)	1.380	±	3.247	3.683	±	7.801	<0.0001
IL6 (pg/mL)	5.130	±	4.403	10.880	±	22.255	0.0141
IL8 (pg/mL)	10.967	±	6.210	15.134	±	11.284	0.0018
Leptin (ng/mL)	3.874	±	2.941	9.107	±	13.380	0.0003
Adiponectin (μg/mL)	41.826	±	59.569	25.253	±	39.202	0.0233
TGFB1 (pg/mL)	94.880	±	285.200	52.659	±	90.076	0.1671

CAD: coronary artery disease; HsCRP: high-sensitivity C-reactive protein; IL6: interleukin 6; IL8: interleukin 8; TGFB1: transforming growth factor beta-1.

**Table 3:** Comparison of body measurements between study groups.

	No CAD		CAD		*p* Value
	Means ± std.		Means ± std.		
Whole body					
Height (cm)	167	±8.422	164.8	±7.682	0.0577
Weight (kg)	66.502	±12.599	72.063	±13.134	0.0028
Body mass index (kg/m^2^)	23.714	±3.299	26.421	±3.720	<0.0001
Waist hip ratio (WHR)	0.895	±0.059	0.941	±0.048	<0.0001
Waist height ratio (WHtR)	0.52	±0.059	0.583	±0.062	<0.0001
Waist thigh ratio (WTR)	2.684	±1.261	1.853	±0.157	<0.0001
Head and neck					
Head surface (cm^2^)	1527	±125.5	1534.7	±145.2	0.6929
Head volume (cm^3^)	5316.4	±591.2	5484.3	±585.0	0.0471
Neck circumference (cm)	41.759	±4.214	43.252	±4.140	0.0132
Trunk					
Chest width (cm)	33.03	±3.522	34.266	±3.191	0.0108
Chest circumference (cm)	97.22	±8.933	103.8	±8.262	<0.0001
Chest sectional area (cm^2^)	7020.2	±1156.1	7585.9	±1212.3	0.0011
Waist width (cm)	31.831	±3.257	33.649	±3.184	0.0001
Waist circumference (cm)	87.212	±11.060	95.92	±9.798	<0.0001
Waist sectional area (cm^2^)	6742.6	±1247.3	7277.4	±1572.8	0.009
Trunk surface area (cm^2^)	7556.9	±1120.9	7919.1	±1180.0	0.0287
Trunk volume (cm^3^)	45,278.3	±9732.4	49,391.8	±9890.0	0.0037
Hip					
Hip width (cm)	35.711	±2.814	35.685	±2.512	0.9456
Hip circumference (cm)	96.699	±8.869	101.9	±8.052	<0.0001
Upper limbs					
Arm length (cm)					
Left	52.766	±3.399	50.677	±3.904	<0.0001
Right	52.917	±3.441	50.73	±3.869	<0.0001
Upper arm circumference (cm)					
Left	30.331	±2.954	31.711	±3.245	0.0021
Right	30.383	±3.036	31.624	±3.377	0.0074
Forearm circumference (cm)					
Left	21.631	±2.778	22.885	±2.966	0.0026
Right	21.973	±2.884	23.065	±2.962	0.0096
Arm surface area (cm^2^)					
Left	1204.3	±146.2	1238.4	±146.8	0.1042
Right	1248.9	±151.8	1279.3	±154.8	0.1656
Arm volume (cm^3^)					
Left	2082.9	±354.0	2160.1	±408.5	0.159
Right	2098.9	±355.2	2213	±447.8	0.0497
Lower limbs					
Thigh circumference (cm)					
Left	51.811	±4.319	51.855	±4.298	0.9438
Right	51.852	±4.248	51.855	±4.379	0.9963
Leg length (cm)					
Left	70.079	±4.532	68.516	±5.047	0.0237
Right	70.129	±4.448	68.441	±4.803	0.0115
Calf circumference (cm)					
Left	31.533	±5.267	31.542	±4.661	0.9907
Right	31.265	±4.171	31.773	±4.751	0.4271
Knee circumference (cm)					
Left	39.631	±3.773	40.17	±4.313	0.3536
Right	39.797	±3.810	40.119	±4.473	0.5878
Leg surface area (cm^2^)					
Left	2822.1	±546.9	2808.2	±496.3	0.8526
Right	2836.3	±538.0	2822.8	±512.9	0.8577
Leg volume (cm^3^)					
Left	6301.1	±1382.5	6399.2	±1444.6	0.6276
Right	6265.4	±1416.0	6411.3	±1427.4	0.4734

CAD: coronary artery disease.

**Table 4:** Association between body measurements and biomarkers.

		HsCRP (mg/L)	IL6 (pg/mL)	IL8 (pg/mL)	Leptin (ng/mL)	Adiponectin (μg/mL)	TGFB1 (pg/mL)
Whole body							
Height (cm)	r	−0.13718	0.01811	0.07323	−0.16755	−0.10611	−0.07586
	*p* value	0.0552	0.8021	0.3102	0.0195	0.1409	0.2931
Weight (kg)	r	−0.03163	0.0954	0.17338	0.06635	−0.24858	−0.14674
	*p* value	0.6599	0.1858	0.0156	0.358	0.0005	0.0412
Body mass index (kg/m^2^)	r	0.04459	0.11043	0.16908	0.1982	−0.24935	−0.14787
	*p* value	0.5349	0.1253	0.0184	0.0056	0.0005	0.0396
Waist hip ratio (WHR)	r	0.09879	0.00763	0.09921	0.19655	−0.27043	−0.20724
	*p* value	0.1683	0.9159	0.1687	0.006	0.0001	0.0037
Waist height ratio (WHtR)	r	0.11197	0.09778	0.12999	0.25614	−0.16595	−0.09625
	*p* value	0.1182	0.175	0.0708	0.0003	0.0207	0.1819
Waist thigh ratio (WTR)	r	−0.09832	0.05043	0.05547	−0.084	0.63179	0.21122
	*p* value	0.1704	0.485	0.4424	0.2443	<0.0001	0.0031
Head and neck							
Head surface (cm^2^)	r	−0.08494	0.0846	0.19626	0.13819	0.15984	−0.01762
	*p* value	0.2366	0.2408	0.0061	0.0547	0.026	0.8074
Head volume (cm^3^)	r	−0.10987	0.1272	0.17475	0.24607	0.14232	−0.03866
	*p* value	0.1253	0.0772	0.0148	0.0005	0.0477	0.5925
Neck circumference (cm)	r	0.02111	0.03145	0.13184	−0.00705	−0.21413	−0.10159
	*p* value	0.769	0.6633	0.0669	0.9223	0.0027	0.1587
Trunk							
Chest width (cm)	r	0.0262	0.10839	0.21173	0.08792	−0.10738	−0.12126
	*p* value	0.7154	0.1325	0.003	0.2228	0.1362	0.0921
Chest circumference (cm)	r	0.0235	0.11379	0.19721	0.18524	−0.13333	−0.16592
	*p* value	0.7437	0.1141	0.0058	0.0097	0.0638	0.0208
Chest sectional area (cm^2^)	r	−0.01108	0.09529	0.18221	0.08779	−0.12291	−0.07173
	*p* value	0.8775	0.1863	0.011	0.2235	0.0878	0.3203
Waist width (cm)	r	0.05531	0.08967	0.19375	0.13998	−0.14666	−0.17349
	*p* value	0.4413	0.2137	0.0068	0.0516	0.0413	0.0156
Waist circumference (cm)	r	0.05435	0.09742	0.1501	0.18334	−0.21226	−0.11769
	*p* value	0.4493	0.1766	0.0367	0.0105	0.003	0.1022
Waist sectional area (cm^2^)	r	0.00772	0.07819	0.19018	0.12382	−0.05133	−0.07408
	*p* value	0.9145	0.2785	0.0079	0.0854	0.4772	0.3047
Truck surface area (cm^2^)	r	0.00952	0.03845	0.14888	0.09488	−0.02907	−0.07203
	*p* value	0.8947	0.5945	0.0383	0.1882	0.6874	0.3183
Truck volume (cm^3^)	r	−0.05931	0.0808	0.15553	0.07081	−0.21734	−0.09545
	*p* value	0.4089	0.2627	0.0304	0.3265	0.0023	0.1855
Hip							
Hip width (cm)	r	−0.055	0.01265	0.08126	0.03089	0.0362	−0.10802
	*p* value	0.4439	0.8611	0.26	0.669	0.6163	0.1338
Hip circumference (cm)	r	0.00261	0.14663	0.1686	0.11914	−0.08771	−0.02172
	*p* value	0.971	0.0413	0.0188	0.098	0.2239	0.7637
Upper limbs							
Arm length (cm)							
Left	r	−0.07267	−0.11303	−0.13079	−0.23207	−0.14424	−0.0883
	*p* value	0.3114	0.1166	0.0691	0.0011	0.0448	0.2208
Right	r	−0.06768	−0.12305	−0.13273	−0.23011	−0.13388	−0.08655
	*p* value	0.3459	0.0874	0.065	0.0012	0.0627	0.2301
Upper arm circumference (cm)							
Left	r	0.01184	0.05659	0.15481	0.14534	−0.09839	−0.12129
	*p* value	0.8692	0.4332	0.0311	0.0432	0.1723	0.0921
Right	r	−0.00758	0.03127	0.16224	0.14506	−0.13694	−0.11651
	*p* value	0.916	0.6651	0.0238	0.0436	0.0569	0.1057
Forearm circumference (cm^2^)							
Left	r	−0.04892	0.11434	0.17707	0.03087	−0.33442	−0.16539
	*p* value	0.4959	0.1124	0.0135	0.6691	<0.0001	0.0212
Right	r	−0.06589	0.06946	0.16128	0.01246	−0.36135	−0.16901
	*p* value	0.3589	0.3358	0.0247	0.8631	<0.0001	0.0185
Arm surface area (cm^2^)							
Left	r	0.09348	0.05002	0.01059	−0.03262	−0.37273	−0.19753
	*p* value	0.1925	0.4885	0.8835	0.6516	<0.0001	0.0058
Right	r	0.02712	0.00412	0.07986	−0.0347	−0.3536	−0.17611
	*p* value	0.706	0.9546	0.2683	0.631	<0.0001	0.014
Arm volume (cm^3^)							
Left	r	−0.01615	0.06738	0.01326	0.05721	−0.18919	−0.11855
	*p* value	0.8222	0.3506	0.8544	0.4282	0.0082	0.0997
Right	r	−0.03485	0.06145	0.13408	0.04729	−0.21427	−0.10349
	*p* value	0.6278	0.3947	0.0623	0.5126	0.0027	0.151
Lower limbs							
Thigh circumference (cm)							
Left	r	−0.05921	0.05597	0.16145	0.11974	0.1302	−0.04956
	*p* value	0.4097	0.4383	0.0245	0.0963	0.0704	0.4925
Right	r	−0.05103	0.04722	0.16389	0.11624	0.16344	−0.04332
	*p* value	0.4775	0.5132	0.0224	0.1065	0.0228	0.5487
Leg length (cm)							
Left	r	0.00066	0.02189	0.02015	−0.12793	−0.09456	−0.07592
	*p* value	0.9927	0.7619	0.7804	0.0755	0.1897	0.2928
Right	r	0.00283	0.01332	0.01618	−0.1234	−0.08464	−0.06908
	*p* value	0.9686	0.8538	0.8228	0.0865	0.2406	0.3385
Calf circumference (cm)							
Left	r	−0.1081	0.09277	0.14462	0.02851	−0.0768	−0.04167
	*p* value	0.1315	0.1982	0.0442	0.6931	0.2871	0.564
Right	r	−0.10161	0.09043	0.1495	0.05331	−0.04868	−0.04637
	*p* value	0.1565	0.2099	0.0375	0.4603	0.5003	0.5209
Knee circumference (cm)							
Left	r	−0.02025	0.0434	0.09408	0.22329	0.13504	−0.12404
	*p* value	0.7781	0.548	0.192	0.0018	0.0605	0.0848
Right	r	−0.06809	0.02673	0.09492	0.21495	0.13828	−0.11538
	*p* value	0.343	0.7115	0.188	0.0026	0.0545	0.1092
Leg surface area (cm^2^)							
Left	r	−0.04733	−0.00004	0.09206	0.19307	0.23322	−0.06176
	*p* value	0.5101	0.9996	0.2017	0.007	0.0011	0.3923
Right	r	−0.05113	−0.01193	0.0916	0.20009	0.24986	−0.04194
	*p* value	0.4767	0.8689	0.204	0.0052	0.0004	0.5615
Leg volume (cm^3^)							
Left	r	−0.05158	0.03393	0.10165	0.19486	0.13742	−0.06532
	*p* value	0.4728	0.6385	0.1584	0.0065	0.056	0.3655
Right	r	−0.04851	0.02795	0.08977	0.2046	0.14678	−0.06536
	*p* value	0.4996	0.6989	0.2132	0.0042	0.0411	0.3652

**Table 5:** Multiple logistic regression analysis of developing CAD.

	Biomarkers				Body Measurements				Biomarkers and Body Measurements			
	Multivariable Logistic Regression Analysis †				Multivariable Logistic Regression Analysis †				Multivariable Logistic Regression Analysis †			
	ORs	Lower	Upper	*p* Value	ORs	Lower	Upper	*p* Value	ORs	Lower	Upper	*p* Value
Chest width (cm)					0.761	0.586	0.987	0.0399	0.62	0.441	0.872	0.0061
Right arm length (cm)					0.743	0.632	0.875	0.0004	0.738	0.603	0.904	0.0033
Waist circumference (cm)					1.119	1.035	1.21	0.0048	1.15	1.038	1.275	0.0078
Leptin (ng/mL)	1.443	1.184	1.76	0.0003					1.504	1.197	1.89	0.0005
Adiponectin (μg/mL)	0.978	0.963	0.994	0.006					0.976	0.959	0.994	0.0094
IL6 (pg/mL)	1.181	1.021	1.366	0.0254					1.256	1.033	1.528	0.0224

CAD: coronary artery disease. †: Adjusted for sex, age, education, exercise, smoking, alcohol and coffee intake, hypertension, diabetes, and hyperlipidemia.

**Table 6:** Area under the receiver operating characteristic curve (AUROC) and 95% confidence interval (CI) for the different scores.

	AUROC	95%CI		*p* Value
Biomarkers score †	0.7492	(0.6812	0.8172)	<0.0001
Body measurements score †	0.7476	(0.6791	0.8161)	<0.0001
Biomarkers and body measurements score †	0.8056	(0.7450	0.8662)	<0.0001
Biomarkers score = (−1.9727) + 0.3669 × Leptin + (−0.0218) × Adiponectin + 0.1661 × IL6 Body measurements score = 13.0994 + (−0.2735) × CW + 0.1125 × WC + (−0.2967) × RAL Biomarkers and body measurements score = (15.3447) + 0.4082 × Leptin + (−0.0239) × Adiponectin + 0.2282 × IL6 + (−0.4781) × CW + 0.14 × WC + (−0.3034) × RAL				

† Adjusted for sex, age, education, exercise, smoking, alcohol and coffee intake, hypertension, diabetes and hyperlipidemia.

## Data Availability

Due to ethical restrictions, the data presented in this study are available on request from the corresponding author.
